# Interspecific comparison of sensitivity to paralytic compounds

**DOI:** 10.17912/micropub.biology.000185

**Published:** 2019-12-17

**Authors:** Vivian Vy Le, Bryan Sanchez, Ray L Hong

**Affiliations:** 1 Department of Biology, California State University, Northridge, 18111 Nordhoff St. Northridge, CA 91330-8303, USA

**Figure 1:**
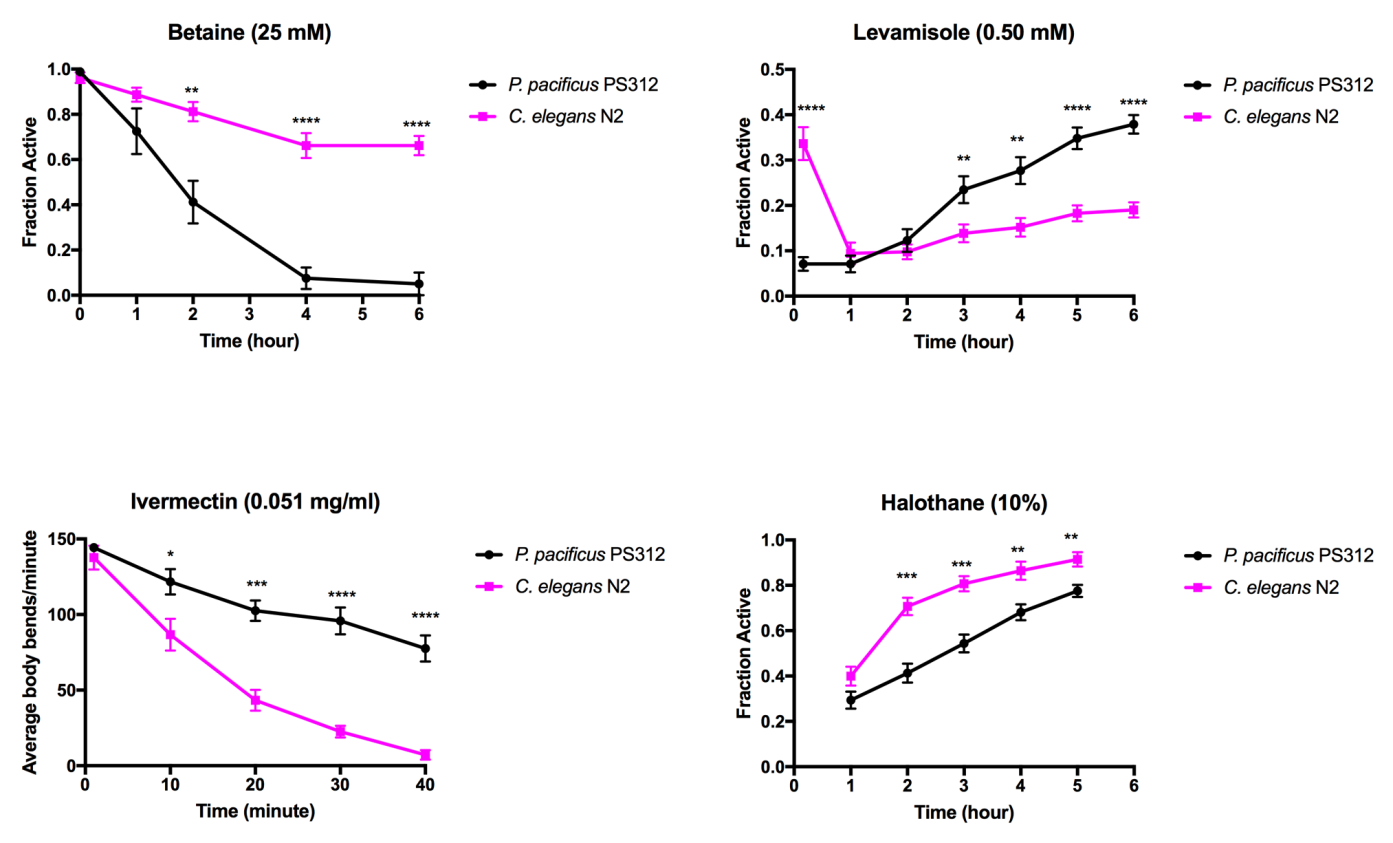
Paralysis was assessed by body movements. Twenty worms were tested per assay and >10 replicates were included per condition. The first reading was taken at 10 minutes for the levamisole and ivermectin treatments. Error bars denote standard error of the mean. Each data set was tested for normality before testing for significance. Unpaired t-test was used to calculate for statistical significances between *P. pacificus* and *C. elegans* for betaine, levamisole, and halothane. The Wilcoxon test was used to calculate for statistical significances for ivermectin. P* < 0.05, P** < 0.01, P*** < 0.001, P**** < 0.0001.

## Description

Identifying the genetic loci conferring resistance to anthelmintic compounds is of paramount importance in assessing and monitoring their effectiveness in the long term. Although *Caenorhabditis elegans* has been a useful reference point for research in parasitic nematodes, a comparative approach using additional genetically tractable nematodes could uncover other unknown factors that contribute to drug sensitivity. To better understand how diverse non-parasitic nematodes respond to known paralytic compounds with anthelmintic activity, we compared for the first time the response of an entomophilic nematode *Pristionchus pacificus* and *C. elegans* to betaine, levamisole, and ivermectin. In addition to paralytic compounds that require physical contact, we also examined the responses toward the volatile anesthetic halothane.

Betaine, a glycine derived amino acid, acts through the acetylcholine receptor ACR-23 expressed in the body muscles and neurons (Peden et al., 2013). The constant activation of the receptor in the presence of excessive exogenous betaine leads to hypercontraction in the muscles and the nervous system, which eventually results in death (Peden et al., 2013). We found that at 25 mM betaine delivered via the medium, almost every *P. pacificus* became paralyzed after four hours compared to ~30% of *C. elegans*.

Levamisole is an agonist for nicotinic acetylcholine receptors expressed in the neuromuscular junctions (nAChR): UNC-38, UNC-39, UNC-63, and LEV-1 (Lewis et al., 1980, Culetto et al., 2004). In *C. elegans*, loss-of-function mutations in several AChR subunits leads to reduced sensitivity to levamisole (Fleming et al., 1997). In vitro experiments have shown that Hco-ACR-8 is critical in levamisole sensitivity in *Haemonchus contortus* and could be a key component in other parasitic nematodes (Blanchard et al., 2018). We found that 0.50 mM levamisole caused ~90% of *C. elegans* and *P. pacificus* to become paralyzed after one hour. Interestingly, *P. pacificus* reached 90% paralysis within ten minutes of treatment, while *C. elegans* took between 10 minutes to an hour to reach the same percentage of paralysis. Although the initial paralysis of *P. pacificus* occurred faster than *C. elegans,* over the course of 6 hours *P. pacificus* was able to recover faster than *C. elegans*.

Ivermectin causes damage to the pharynx that lead to paralysis, starvation, and death by inhibiting pharyngeal pumping. Ivermectin targets *avr-14*, *avr-15*, and *glc-1* that encode glutamate chloride subunit channels (Dent et al., 1999). In *C. elegans*, the loss of function in glutamate-gated chloride channel subunits results in resistance to several avermectins (Ghosh et al., 2012). Rescuing the *avr-14* mutant with Hco-AVR-14B cDNA from *Haemonchus contortus* restored sensitivity towards ivermectin, suggesting that orthologous gene products from parasitic nematodes can function in *C. elegans* (Glendinning et al., 2011). We found that in liquid swim assays with 0.051 mg/ml ivermectin, *C. elegans* became paralyzed faster than *P. pacificus*.

Halothane, a volatile anesthetic, binds to the syntaxins and SNARE complex and acts on neurotransmitters (Nagele et al., 2005). We found that while most worms in both nematode species were paralyzed within the first hour, *C. elegans* recovered faster than *P. pacificus.*

Taken together, *P. pacificus* is more sensitive to betaine and halothane than *C. elegans*, while *C. elegans* is more sensitive to levamisole and ivermectin than *P. pacificus*. However, *P. pacificus* seems to recover faster than *C. elegans* after two hours in the presence of levamisole, suggesting a possible divergence in nicotinic receptors. These results point to significant differences between the two species in drug uptake, receptor sensitivity, and metabolism.

## Methods

Nematodes were cultured on OP50 *E. coli* and NGM media at 20°C. 20 J4 *P. pacificus* or L4 *C. elegans* were picked from a non-starved culture and transferred onto an unseeded plate or liquid buffer containing the drug compound at 20°C for the duration of the experiment.

2.5 M Betaine hydrochloride solution was made with deionized nuclease-free water and stored in 4°C. Betaine was top plated onto 35 mm NGM plates containing ~6 ml of agar by spreading 60 μl of the stock solution and left to dry overnight. 0.10 M Levamisole stock solution was made with deionized nuclease-free water. Stock solutions were stored in -20°C. Levamisole was added to NGM media without cholesterol before being poured: ~6 ml of agar into 35 mm plates. For betaine and levamisole assays, the number of active and paralyzed worms were tallied every hour for 6 hours.

A 25 mg/ml ivermectin stock solution (28.57 mM) was diluted in 100% ethanol and stored in -20°C. Twenty L4 or J4 worms were individually transferred into a 96-well flat-bottom tissue culture plates (Cellstar 655 180). Each well contained 100 μl of 1:500 dilutions of ethanol or ivermectin in Nanopure water (58 µM ivermectin). Movements were tallied visually for 10 seconds twice in one minute for every ten-minute interval for forty minutes. Movements were tallied when a worm made a full wave of the body and/or bending to the dorsal or ventral side of the midsection. At each time interval, moving bodies were counted for ten seconds and twice within a minute. Worms that lacked movement during the first two counts were disregarded as injured by the transfer. The counts of movement per 10 seconds were averaged between the two counts and multiplied by six to tally the body movement per minute for each worm.

10% Halothane solution was diluted in 100% ethanol in 1 ml tubes covered with aluminum foil under the fume hood. Twenty L4 or J4 worms were individually picked from seeded plates and transferred onto an unseeded 35 mm NGM plate. Under the fume hood with the lights off, 10 μl of diluted halothane is added to a small piece of Whatman filter paper made by a standard office hole-puncher and placed on the lid. The dish was parafilmed immediately, covered with aluminum foil, and stored in 20°C for the duration of the experiment. The number of active and paralyzed worms were tallied every hour for 6 hours.

Prism by GraphPad (v7.0d, San Diego, CA) was used for graphical and statistical analysis.

## Reagents

Betaine hydrochloride (Acros Organics 164552500); Ivermectin (Alfa Aesar J6277); Levamisole hydrochloride (Acros Organics 187870100); Halothane: 2-bromo-2-chloro-1,1,1-trifluoethane (Sigma-Aldrich B4388). The wildtype nematode strains are *Caenorhabditis elegans* N2 and *Pristionchus pacificus* RS2333 (a laboratory derivative of the original strain PS312).
