# Corrigendum: Interspecific comparison of sensitivity to paralytic compounds

**DOI:** 10.17912/micropub.biology.000238

**Published:** 2020-04-01

## Description

For Le, VV; Sanchez, B; Hong, RL (2019). Interspecific comparison of sensitivity to paralytic compounds. microPublication Biology. 10.17912/micropub.biology.000185

The paralytic in the following sentence is incorrect:

“However, *P. pacificus* seems to recover faster than *C. elegans* after two hours in the presence of ivermectin, suggesting a possible divergence in nicotinic receptors. ”

The authors correct the sentence to read:

“However, *P. pacificus* seems to recover faster than *C. elegans* after two hours in the presence of levamisole, suggesting a possible divergence in nicotinic receptors. ”

This error has been corrected online.

